# Essential Elements and Their Relations to Phenolic Compounds in Infusions of Medicinal Plants Acquired from Different European Regions

**DOI:** 10.1007/s12011-015-0481-6

**Published:** 2015-08-27

**Authors:** P. Konieczynski, A. Arceusz, M. Wesolowski

**Affiliations:** Department of Analytical Chemistry, Medical University of Gdansk, Gen. J. Hallera 107, 80-416 Gdansk, Poland

**Keywords:** Essential elements, Phenolic compounds, Antioxidant activity, Medicinal herbs, Statistical methods

## Abstract

The aim of this research was to compare chemical composition of herbs acquired from different European countries. The concentrations of P, Fe, Mn, Zn, Cu, phenolic compounds, and the antioxidant activity were determined in infusions of 27 medicinal herbs (7 species) from Lithuania, Serbia, Italy, and Portugal. Total and extractable P were expressed in milligrams per liter and metals in micrograms per liter and followed the sequence: Fe > Mn > Zn > Cu, while antioxidant activity ranged from 29.4 to 217.8 mg of Trolox equivalent (TE) per liter. Total flavonoids were in the range of 20.5–95.1 mg L^−1^. The rank order of phenolic compounds assayed by HPLC method (in mg L^−1^ of infusion) was as follows: rutin > myricetin > quercetin > kaempferol, and chlorogenic > ferulic > *p*-coumaric > caffeic > gallic acids. Significant correlations were found between total P–inorganic phosphate P, Zn–Mn, Mn–Cu, total flavonoids–antioxidant activity, and quercetin with caffeic and ferulic acids. Generally, medicinal plant infusions differed in their chemical composition, strongly depending on plant species, regardless of the origin from distant geographical areas of Europe. Principal component analysis selected the concentrations of Cu, Mn, total and inorganic phosphate P, as factors which strongly influence differentiation of the samples. Moreover, infusions from *Hyperici herba* and *Helichrysi inflorescentia* contained significant amounts of water-extractable Mn and Fe forms as claimed by the Dietary Reference Intakes for humans.

## Introduction

Herbal medicinal products are still in focus of researchers worldwide and the consumption of herbal preparations, even in highly developed countries, is high [1–3]. In this situation, there is a necessity to monitor chemical composition of herbal preparations. Moreover, by studying the chemical composition of infusions (*infusa*), decoctions (*decocta*), macerates (*macerata*), herbal teas (*plantae ad ptisanam*), and herbal tea mixtures (*species ad ptisanam*), it would be possible to learn which chemical forms of the elements are bioavailable for humans, when using these herbal preparations.

So far, numerous secondary metabolites in herbal medicinal products have been thoroughly studied, including their fingerprint analysis [4–6]. In recent years, the need for standardization of herbal preparations is growing [7, 8]. Therefore elemental composition of herbal drugs representing different plant species, as well as various morphological plant organs, was investigated [9–11]. Several studies were performed to establish the influence of environment and/or botanical plant species on the chemical composition, including the levels of metallic and non-metallic elements in herbal medicinal products [12–15].

Phenolic compounds were also studied in various herbal medicinal products, including different types of herbal substances, and in herbal preparations [16–21]. For example, the content of phenolic compounds and the antioxidant activity of herbal infusions from Amazonian region were investigated [16]. It was demonstrated that the studied nine herbs had the polyphenol/flavonoid content and antioxidant properties similar to those of a typical infusion obtained from *Camellia* species. A study on a popular antioxidant herb—*Cistus incanus* has shown that incorrect choice of brewing process parameters can result in decreased polyphenolics content in infusion of that herb [17]. A research on mate tea has revealed that also the way of preparation of herbal infusion has an essential influence on the level of phenolic compounds in the infusion [19].

Several European countries have become important suppliers of herbs used in the pharmaceutical and cosmetics industries. Among them are herbal enterprises located in Southern Europe, for example in Serbia, Bulgaria, Italy, and Portugal, and in the Central and Eastern Europe, including Poland, Ukraine, and Lithuania. Therefore, in this research, a particular emphasis has been laid on chemical composition of herbs originating from distant areas of Europe.

It is commonly known that medicinal plants grow or are cultivated in different climatic conditions, also on various types of soil, climate, and pollution of the environment, so all these factors have a significant impact on plant’s chemical composition represented by typical secondary metabolites, such as flavonoids, phenolic acids and also by essential elements. Therefore, the purpose of this study was to compare the chemical composition of medicinal plants representing seven plant species originating from various areas of Europe (Lithuania, Serbia, Italy, and Portugal), including selected essential elements and phenolic compounds. This comparison should help to answer the question whether or not plants of the same species differ significantly taking into account their origin from various European regions. Therefore, analysis of variance, correlation analysis, and multivariate statistical tools, such as cluster and principal component analyses, were applied. Moreover, the contribution of water-extractable forms of Fe, Zn, Mn, and Cu in infusions of the medicinal herbs to the recommended Dietary Reference Intakes were calculated to indicate the most valuable sources of essential elements.

## Materials and Methods

### Plant Material and Sample Preparation

The analyzed herbal medicinal products originated from herbal companies located in Lithuania, Serbia, Portugal, and Italy, as presented in Table 1. The dried plant materials were ground using a Knifetec 1095 sample mill, sieved through a 0.5-mm mesh sieve. (Foss-Tecator, Denmark), and stored in polyethylene containers until analysis.

**Table 1:** The medicinal plant materials under study

Sample no.	Herbal remedy	Medical use	Plant species, botanical family	Herbal company	Country of origin
1	*Equiseti herba*	Diuretic, supporting in therapy of tuberculosis	*Equisetum arvense* L., *Equisetaceae*	Dr. P. Karvelis	Lithuania
2				Adonis Sanobanja	Serbia
3				Beli gor Svrlijig	Serbia
4				Ervital	Portugal
5				Roma	Italy
6	*Polygoni herba*	Hepatoprotective, in therapy of tuberculosis, diuretic	*Polygonum aviculare* L., *Polygonaceae*	SVF	Lithuania
7				Adonis Sanobanja	Serbia
8	*Hyperici herba*	Antidepressant, for pelvic pain and cramping, anti-inflammatory for strains, sprains, and contusions	*Hypericum perforatum* L., *Guttiferae*	SVF	Lithuania
9				Adonis Sanobanja	Serbia
10				Moc bilja	Serbia
11				Planinska	Serbia
12				Ervital	Portugal
13	*Crataegi folium et flos*	Cardiacum, vasodilatans, anti-arhythmic	*Crataegus oxyacantha* L., *Rosaceae*	Acorus	Lithuania
14				Adonis Sanobanja	Serbia
15				Roma	Italy
16	*Sambuci flos*	Mild diuretic, increases sweatening	*Sambucus nigra* L., *Caprifoliaceae*	Sirdazole	Lithuania
17				Adonis Sanobanja	Serbia
18				Beli gor Svrlijig	Serbia
19				Ervital	Portugal
20				Roma	Italy
21	*Chamomillae flos*	Anti-inflammatory, immunomodulary anti-diabetic, acaricidal, anti-hyperglycemic, anti-microbial	*Matricaria chamomilla* L., *Compositae*	SVF	Lithuania
22				Moc bilja	Serbia
23				Adonis Sanobanja	Serbia
24				Ervital	Portugal
25				Roma	Italy
26	*Helichrysi inflorescentia*	Cholereticum, cholagogum, antihelminticum, antibioticum	*Helichrysum arenarium* L. Moench, *Compositae*	Ervital	Portugal
27				Dr. P. Karvelis	Lithuania

Infusions of medicinal herbs were obtained by pouring boiling redistilled water (100 mL) onto 2.0 g of a plant material. After 15 min, the infusions were filtered through paper filters (Filtrak No 389, Germany) and diluted to 100 mL with redistilled water obtained from a Heraeus (Switzerland) distillation system.

Next, the infusions were filtered through a 0.20-μm nylon membrane filter (Witko Łódź, Poland) into a HPLC vial, as recommended by a procedure for ultracleaning of solvents prior to HPLC analysis. For each sample, the complete assay procedure was repeated in triplicate, and the standard deviation was calculated.

### Reagents

Standards of phenolic acids such as gallic (GA), chlorogenic (CGA), caffeic (CA), *p*-coumaric (*p*CA), ferulic (FA) and of flavonoids such as rutin (RUT), myricetin (M), quercetin (Q), and kaempferol (K) were purchased from ChromaDex (CA, USA). HPLC-grade methanol was purchased from Avantor Performance Materials Poland S.A., and trifluoroacetic acid (TFA) was from Sigma-Aldrich (St. Louis, MO, USA).

### Chromatographic Conditions

Chromatographic analyses were performed using a HPLC LaChrom (Merck, Darmstadt, Germany) system consisting of an L-7100 pomp, an L-7360 column compartment, and an L-7420 UV/vis detector. The chromatographic separation was performed on a Hypersil Gold C18 column (250 × 4.6 mm, i.d. 5 μm) which was placed in a thermostat at 35 °C. The mobile phase was consisted of solvent A (0.05 % TFA in methanol) and solvent B (0.05 % TFA in water). The optimized gradient elution was performed using the following program: 5–25 % A (0–30 min), 25–40 % A (30–40 min), 40–60 % A (40–50 min), and 5 % A (50–60 min). Conditions for sample pretreatment had previously been optimized by studying the type of solvent (methanol, ethanol), solvent concentration in water, and extraction time [4]. Before starting gradient runs, initial conditions were maintained during 10 min for column equilibration. The flow rate was set at 1.0 mL min^−1^ and the injection volume was 20 μL. The wavelength of the UV/vis detector was set at 280 nm for gallic and *p*-coumaric acids; at 320 nm for caffeic, ferulic, and chlorogenic acids; and at 370 nm for flavonoids. The identification of the phenolic compounds in the samples was based on comparison of retention time with those of the standards.

### Spectrophotometric Measurement

For all spectrophotometric measurements, a Metertek SP-870 (South Korea) UV/vis instrument was used. The contents of total flavonoids were directly determined in infusions at 510 nm using the reaction with AlCl_3_ based on the rutin standard (Across Organics, Belgium). Total and extractable phosphorus was determined by spectrophotometric method using a phosphate-molybdate complex (*λ* = 650 nm).

### Radical Scavenging Activity (DPPH Assay)

A spectrophotometric analysis of radical scavenging activity using the DPPH method with a Trolox calibration curve was performed. This assay is based on the ability of the antioxidant to scavenge the radical cation DPPH. Ten microliters of the infusion were added to 2 mL of methanolic DPPH (0.04 mmol L^−1^). After 60 min of incubation in a dark place at room temperature, the absorbance was measured at 517 nm using 10-mm quartz cuvettes. A calibration curve in the range 0.1–1.0 mmol L^−1^ was used for the Trolox, and the data were expressed as a Trolox equivalent antioxidant capacity (TEAC, mg L^−1^).

### Metallic Elements Determination

The essential metallic elements were assayed using standard analytical conditions (air/acetylene), applying a flame program of the Atomic Absorption Spectrometer 250 Plus (Varian, Australia) and the following analytical wavelengths (nm): 248.3 (Fe), 213.9 (Zn), 279.5 (Mn), and 324.8 (Cu).

### Validation of the Analytical Methods

The validation of the methods included calculation of values of regression equations for calibration curves with *S*_a_ and *S*_b_, limits of detection (LOD) and quantification (LOQ), as shown in Table 2. By assuming the “*r*” values higher than 0.99, the linearity obtained for the assayed elements and other analytes, as well as for the LOD and LOQ values, they were recognized as being on an acceptable level.

**Table 2:** Validation parameters of the developed analytical procedures for quantification of essential elements and phenolic compounds

Analyte	Range (μg mL^−1^)	Regression equation	Parameter of validation				
			*S*_a_	*S*_b_	Linearity (*r*)	LOD (μg mL^−1^)	LOQ (μg mL^−1^)
P	1.6–8.0	*A* = 0.01670 + 0.10819 × *x*	0.0019	0.0102	0.9988	0.05	0.15
Fe	1.0–5.0	*A* = 0.0014 + 0.03620 × *x*	0.0009	0.0030	0.9991	0.05	0.16
Zn	1.0–4.0	*A* = 0.09750 + 0.14730 × *x*	0.0147	0.0400	0.9901	0.01	0.04
Mn	1.0–5.0	*A* = 0.02250 + 0.10670 × *x*	0.0050	0.0169	0.9965	0.02	0.05
Cu	0.2–1.2	*A* = −0.0073 + 0.08980 × *x*	0.0040	0.0030	0.9965	0.02	0.06
Rutin	100–500	*Y* = 64,500 + 11,760 × *x*	1421	65,104	0.9857	27.26	82.61
Myricetin	100–500	*Y* = −2,750,000 + 41,770 × *x*	1468	410,277	0.9987	21.42	64.93
Quercetin	100–500	*Y* = −2,720,000 + 44,402 × *x*	1501	419,604	0.9988	19.80	60.00
Kaempferol	100–500	*Y* = −8,020,000 + 138,100 × *x*	5941	1,660,545	0.9981	22.27	67.48
Gallic acid	10–100	*Y* = 3045*x* + 40,643	129	7835	0.9946	0.34	1.11
Chlorogenic acid	10–100	*Y* = 16759*x* + 45,609	474	28,777	0.9976	1.32	4.02
*p*-Coumaric acid	10–100	*Y* = 11974*x* + 256,233	354	21,456	0.9974	1.40	4.89
Caffeic acid	10–100	*Y* = 1329*x* + 50,758	91	6432	0.9953	0.76	2.95
Ferulic acid	10–100	*Y* = 6771*x* + 67,313	359	21,780	0.9916	0.95	1.96

*S*_*a*_ standard deviation of the slope, *S*_*b*_ standard deviation of the intercept, *LOD* limit of detection, *LOQ* limit of quantification

As for HPLC determinations, the linearity was examined with standard solutions. Mixed stock solutions (1 mg mL^−1^) of nine phenolic compounds were prepared. Each calibration curve was based on five different concentrations of a phenolic compound. Calibration working standards were freshly prepared in methanol by appropriate dilution of the stock solutions. The linearity for each phenolic compound was established by plotting the peak area (*Y*) against concentration (*X*) of each compound and had been verified by a correlation study. In this way, calibration curves of nine phenolic compounds were obtained. The LODs and LOQs expressed by 3- and 10-fold signal-to-noise (S/N) ratios were also obtained.

### Data Analysis

Statistical analyses such as one-way analysis of variance (ANOVA) and the correlation, cluster (CA), and principal component analyses (PCA) were calculated using a *Statistica 7.1* (Statsoft, Poland) software [22]. ANOVA helped to determine statistically significant differences between the analyzed samples due to their origin from different botanical species and from distant areas of growth in Europe. Correlation analysis was used in order to reveal the interrelations among the essential elements and phenolic compounds, whereas multivariate statistical methods helped to find the patterns, in which the studied samples were clustered, and in the case of PCA, this method enabled to reduce the multidimensionality of the experimental database.

## Results and Discussion

### The Contents of Macro- and Trace Elements

The highest level of all studied elements represented total phosphorus—96.3 mg L^−1^. However, in particular samples, its level was differentiated, ranging from 36.7 in *Equiseti herba* to 184.7 mg L^−1^ in *Sambuci flos*. Taking into consideration the mean concentration of total P in different plant species, the highest amounts of this essential element was found in *S. flos* and in *Chamomillae flos*, 146.8 and 124.2 mg L^−1^, respectively. The lowest level was determined in the species of *Helichrysi flos*—56.4 mg L^−1^. The levels of water-extractable P in the infusions were about 50 % lower than those of the total P and ranged from 17.1 in *E. herba* to 87.9 mg L^−1^ in *S. flos*.

In the group of metallic elements, the highest mean concentration of Fe was found in infusion—6373.6 μg L^−1^. Next, Mn can be listed, with the mean of 2102.7 μg L^−1^. The mean concentration of Zn was found as 966.4 μg L^−1^, and the lowest of Cu—75.8 μg L^−1^.

The range of Fe in the studied samples was from 1703.3 in *E. herba* to 15,848.2 μg L^−1^ in *C*. *flos*. For Mn, the range of concentrations was found from 191.7 in *E. herba* to 4862.3 μg L^−1^ in *Hyperici herba*. The amount of Zn was determined within the range from 272.7 in *E*. *herba* to 1622.3 μg L^−1^ in *Helichrysi inflorescentia*, and the range of Cu from 28.3 in *Polygoni herba* to 261.0 μg L^−1^ in *E. herba*. These values are similar to those reported earlier for medicinal plants [9–15].

### Total Contents of Phenolic Compounds and the Antioxidant Activity

All results are presented in Table 3. The mean concentration of total flavonoids in infusions was established as 54.7 mg L^−1^ with the standard deviation of 1.9 mg L^−1^. The lowest level was determined in *P. herba*—20.5 mg L^−1^, whereas the highest in *H. herba*—95.1 mg L^−1^. High total flavonoid content was also found in other samples of *Hyperici herba*, from 71.4 to 124.4 mg L^−1^. The obtained results of total flavonoid content are on similar level, as reported in the literature [16–21]. For example, the antioxidant potential of herbs collected in Brazilian Amazonian region was found between 4.2 and 43.8 mg L^−1^, and also for these plant materials, this value was strongly dependent on the botanical species of the herb [16]. During another study, it was found in extracts of a plant growing in Serbia, *Marrubium peregrinum*, that total flavonoid concentration was within the range 18.7–54.8 mg g^−1^ [21]. Our results fall in a similar range. The antioxidant activity ranged from 29.4 to 217.8 mg of Trolox equivalent (TE) per liter and it depended on plant species.

**Table 3:** The content of analytes in the herbal infusions. Range, median, arithmetic mean ± SD and RSD (%) are shown

	*Equiseti herba* (*n* = *5*)	*Polygoni herba* (*n* = *2*)	*Hyperici herba* (*n* = *5*)	*Crataegi folium et flos* (*n* = *3*)	*Sambuci flos* (*n* = *5*)	*Chamomillae flos* (*n* = *5*)	*Helichrysi inflorescentia* (*n* = *2*)
P	14.9–33.1, 18.6; 22.5 ± 7.7; 34.2	17.0–33.0, 25.1; 25.1 ± 11.4; 45.4	23.4–34.0, 28.9; 28.0 ± 4.0; 14.3	28.0–30.9, 29.5; 29.5 ± 1.4; 4.8	36.6–58.4, 58.1; 53.5 ± 9.5; 17.8	37.4–63.8, 54.0; 50.8 ± 10.4; 20.5	23.6–31.5, 27.6; 27.6 ± 5.6; 20.3
P–PO_4_	8.5–22.3, 11.6; 13.3 ± 5.4; 40.6	12.8–27.7, 20.3; 20.3 ± 10.5; 51.7	14.8–31.0, 22.0; 21.3 ± 6.2; 29.1	23.1–27.9, 23.4; 24.8 ± 2.7; 10.9	33.3–44.0; 36.9 ± 4.4; 11.9	22.9–43.1, 31.4; 32.4 ± 7.4; 22.8	18.1–21.8, 19.9; 19.9 ± 2.6; 13.1
Fe	689.5–4868.8, 1447.4; 2549.5 ± 1935.2; 75.9	2009.5–5710.2, 3859.8; 3859.8 ± 2616.8; 67.8	617.9–3430.0, 832.2; 1339.4 ± 1178.1; 88.0	1030.5–2599.5, 1740.2; 1790.1 ± 785.7; 43.9	1111.4–4130.3, 1897.6; 2065.5 ± 1215.4; 58.8	1410.0–5138.8, 1929.3;2601.1 ± 1567.8; 60.3	1056.0–1165.9, 1111.0; 1111.0 ± 77.7; 7.0
Zn	79.9–297.9, 273.9; 236.9 ± 89.8; 37.9	255.9–404.8, 330.4; 330.4 ± 105.2; 31.8	286.2–482.0368.6; 373.1 ± 76.0; 20.4	242.0–392.9 351.3; 328.7 ± 77.9; 23.7	329.3–392.8 335.6; 350.4 ± 27.3; 7.8	283.2–440.2 326.0; 339.7 ± 61.9; 18.2	570.1–656.7 613.4; 613.4 ± 61.3; 10.0
Mn	109.0–426.3, 271.2; 263.7 ± 128.3;48.6	491.4–880.9, 686.1; 686.1 ± 275.4; 40.1	961.1–1716.0, 1212.0; 1270.7 ± 281.8; 22.2	737.1–807.6 741.4; 762.1 ± 39.5; 5.2	576.1–805.7 609.0; 652.1 ± 92.7; 14.2	528.9–1083.9 548.0; 662.4 ± 238.0; 35.9	1203.9–1303.7, 1253.8; 1253.8 ± 70.6; 5.6
Cu	13.1–91.8, 81.0; 68.1 ± 31.7; 46.5	10.8–14.7, 12.8; 12.8 ± 2.8; 21.9	11.3–19.0, 14.2; 15.3 ± 3.7; 24.2	18.7–22.2, 19.3; 20.1 ± 1.9; 9.5	19.4–23.2, 21.9; 21.4 ± 1.5; 7.0	25.4–29.8, 26.9; 27.4 ± 1.8; 6.6	32.5–33.5, 33.0; 33.0 ± 0.7; 2.1
Total flavonoids	25.8–54.3, 28.9; 34.2 ± 11.9; 34.8	18.9–22.2, 20.5; 20.5 ± 2.3; 11.2	71.4–124.4, 91.3; 95.1 ± 21.4; 22.5	68.2–73.2, 72.5;71.3 ± 2.7; 3.8	39.9–66.3, 55.2; 55.2 ± 10.4; 18.9	23.7–60.4, 35.1; 37.5 ± 13.8; 36.8	64.5–66.8, 65.6; 65.6 ± 1.62.4
Rutin	7.9–24.7, 13.5; 15.8 ± 6.7; 42.4	nd	94.8–602.5, 218.9; 368.3 ± 393.3; 106.8	149.2–346.8, 238.9; 244.9 ± 98.9; 40.4	446.0–933.4, 596.3; 634.1 ± 179.9; 28.4	nd	188.9–188.9, 188.9; 188.9 ± 0.1; 0.1
Mirycetin	67.6–99.2, 86.7; 85.6 ± 11.4; 13.3	66.6–70.4, 68.5; 68.5 ± 2.7; 4.0	66.1–74.5, 66.3; 69.2 ± 4.1; 6.0	nd	67.1–67.9, 67.4; 67.4 ± 0.3; 0.4	69.9–88.4, 73.8; 76.2 ± 7.1; 9.3	69.1–69.1, 69.1; 69.1 ± 0.1; 0.0
Quercetin	nd	nd	61.3–61.3, 61.3; 61.3 ± 0.1; 0.0	nd	61.4–61.5, 61.4; 61.4 ± 0.1; 0.1	61.5–88.3, 71.5; 73.2 ± 12.6; 17.2	61.6–61.7, 61.7; 61.7 ± 0.1; 0.2
Kaempferol	nd	nd	nd	nd	58.1–58.1, 58.1; 58.1 ± 0.1; 0.0	nd	58.1–58.2, 58.2; 58.2 ± 0.5; 1.0
Gallic acid	2.8–9.7, 4.9; 5.5 ± 2.5; 46.2	7.1–8.6, 7.8; 7.8 ± 1.1; 14.0	3.0–3.4, 3.2; 3.2 ± 0.2; 6.2	2.9–3.2, 2.9; 3.0 ± 0.1; 3.3	3.7–6.7, 4.5; 4.7 ± 1.2; 25.5	2.9–3.5, 3.3; 3.3 ± 0.2; 6.0	3.1–3.6, 3.4; 3.4 ± 0.4; 11.8
Chlorogenic acid	0.2–55.2, 36.4; 33.4 ± 20.2; 60.4	0.7–3.1, 1.9; 1.9 ± 1.7; 89.5	5.7–60.0, 16.0; 30.2 ± 26.3; 87.1	4.6–400.5, 308.6;237.9 ± 207.2; 87.1	2.7–754.9, 47.6; 154.5 ± 33.6; 21.7	2.1–236.5, 71.8; 91.2 ± 99.8; 109.4	7.3–42.6, 24.9; 24.9 ± 24.9; 100.0
Caffeic acid	0.8–8.2, 2.1; 3.6 ± 2.9; 80.5	0.3–0.4, 0.3; 0.3 ± 0.1; 33.3	3.5–10.0, 6.1; 6.1 ± 2.5; 41.0	2.1–2.8, 2.4; 2.4 ± 0.4; 16.7	0.8–3.1, 2.3; 2.1 ± 0.9; 42.9	0.3–82.1, 9.6; 35.3 ± 40.1; 113.6	0.2–7.2, 3.7; 3.7 ± 4.9; 132.4
*p*-Coumaric acid	2.9–85.3, 68.5; 53.1 ± 36.0; 67.8	1.5–1.9, 1.7; 1.7 ± 0.3; 17.6	1.0–2.7, 2.2; 2.1 ± 0.7; 33.3	3.2–9.2, 3.9; 5.4 ± 3.3; 61.1	22.4–55.8, 50.9; 45.9 ± 13.7; 29.9	2.7–3.8, 3.2; 3.2 ± 0.5; 15.6	6.4–7.2, 6.8; 6.8 ± 0.6; 8.8
Ferulic acid	0.6–2.4, 2.2; 1.7 ± 0.8; 47.0	4.5–4.5, 4.5; 4.5 ± 0.1; 0.0	0.4–2.9, 4.5; 1.0 ± 1.2; 120.0	0.9–0.9, 0.9; 9.2 ± 0.1; 0.0	2.1–2.5, 3.8; 8.6 ± 9.7; 112.8	5.9–173.5, 128.2; 114.4 ± 63.5; 55.5	0.2–12.2, 6.1; 6.1 ± 8.5; 139.3
Antioxidant activity	29.4–39.5, 34.7; 34.8 ± 04.4; 12.6	41.0–42.5, 41.7; 41.7 ± 1.1; 2.6	186.2–218.0, 186.9; 193.6 ± 13.6; 7.0	43.1–47.2, 45.5; 45.3 ± 2.1; 4.6	34.6–47.3, 45.5; 42.4 ± 5.5; 13.0	43.9–47.4, 45.7; 45.6 ± 1.3; 2.9	39.0–43.8, 41.4; 41.4 ± 3.4; 8.2

Total and extractable P, total flavonoids, particular flavonoids, and phenolic acids are in mg/L, metallic elements in μg/L, and antioxidant activity in mg of Trolox equivalent/L

*nd* not detected (below LOD)

The content of rutin extended from 7.9 in *E. herba* to 933.4 mg L^−1^ in *S. flos*. The mean concentration of this flavonoid was equal to 330.5 mg L^−1^ of infusion. The highest mean level of rutin was noticed in the infusion obtained from *S. flos*, whereas the lowest was found in *E. herba*. The lowest level of myricetin was determined in *H. herba*, 66.1 mg L^−1^, and the highest in *E. herba*, 99.2 mg L^−1^. The mean concentration of myricetin in all analyzed infusions was 72.7 mg L^−1^. The contents of quercetin ranged from 61.3 in *H. herba* to 88.3 mg L^−1^ in *C. flos*, and the mean concentration of this flavonoid was 64.5 mg L^−1^ in all studied infusions. The content of kaempferol fell in a rather narrow range of concentrations, from 58.1 mg L^−1^ in *S. flos* to 58.2 mg L^−1^ in *H. inforescentia*. By comparison of our results with the literature data, it can be stated that they are compatible with those reported in the literature [16–21].

With the results of phenolic acids assays, which fell in the range of concentration of several milligrams per liter, it is possible to distinguish some characteristic plant samples. For example, high concentration of gallic acid was found in one sample of *E. herba*—9.7 mg L^−1^—and in two of *P. herba*—7.1 and 8.6 mg L^−1^. Quite high levels of chlorogenic, *p*-coumaric, and caffeic acids, above 10.0 mg L^−1^, were determined in *E. herba*, *Crataegi folium et flos*, and in *S. flos*. On the other hand, the highest concentration of ferulic acid was found in *C. flos*—above 173.0 mg L^−1^. As it was reported earlier, phenolic acids in herbal infusions of lemon balm covered concentration range from 0.001 to 1.589 mg g^−1^ of dry weight [4]. It can be concluded that the level of phenolic acids determined in the studied samples of medicinal plants is on a similar level, as reported by other researchers [16–21].

### Factors Influencing the Differences Between the Studied Plant Samples

In order to identify statistically significant differences in concentrations of the analytes, ANOVA was used. Especially important was to learn whether or not these differences were due to the fact that analyzed samples originated from different plant species (genetic factor) or from distant areas of growth (geographical aspect).

Taking into consideration the differences due to the origin of samples, only in the case of two metallic elements, Fe and Zn, they were statistically significant between the samples collected in Serbia and those from Portugal. This was confirmed by the results of a post hoc test of LSD (least significant difference), for which the *p* values were lower than 0.05. However, for the other analytes, the influence of the growth area was not statistically significant.

On the other hand, the results of ANOVA confirmed the statistically significant differences in the level of 13 analytes arising from the fact of origin of studied herbal samples from different botanical species. The most frequent differences occurred between the level of Mn and total flavonoid contents. The reason for this differentiation is the genetic factor, which also confirms earlier findings in relation to analysis of elements in medicinal plant families [23].

### Relationships Among Essential Elements and the Phenolic Compounds

**Fig. 1:**
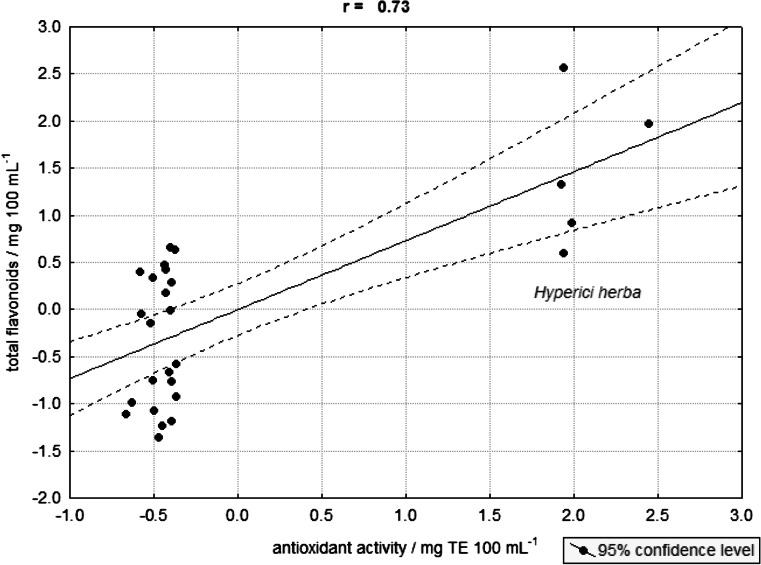
Correlation between the antioxidant activity and total flavonoids content for the medicinal herbs from various European regions

The results of correlation analysis are shown in Table 4. Statistically significant correlations (*α* < 0.05) were found between the pairs: total P and phosphate P, Zn–Mn, Mn–Cu, and total flavonoids–antioxidant activity. The relationship of antioxidant activity vs total flavonoid content is presented in Fig. 1, and it can be noted the characteristic group of samples from one plant species—*Hypericum perforatum*. Several statistically significant relations were also found among phenolic acids and other analytes, for instance, between metallic elements (Zn, Mn and Cu), and *p*-coumaric acid. High correlations were found for caffeic acid–quercetin (*r* = 0.98) and for ferulic acid–quercetin (*r* = 0.81) pairs.

**Table 4:** The results of correlation analysis

	∑ Fl	Rutin	Myricetin	Quercetin	Fe	Mn	Zn	Cu	P	CA
Myricetin	−0.29	−*0.62*	1							
Quercetin	−0.57	0.11	0.27	1						
Kaempferol	0.62	−0.53	0.02	0.41						
Fe	−0.30	0.05	0.07	*0.60*	1					
Mn	*0.73*	0.23	−*0.42*	−0.39	−*0.38*	1				
Zn	0.29	*0.46*	−0.27	−0.23	−0.33	*0.57*	1			
Cu	−0.33	−*0.50*	*0.62*	0.26	−0.18	−*0.59*	−0.35	1		
P	−0.07	*0.59*	−0.19	−0.01	−0.09	−0.08	0.06	−0.30	1	
P–PO_4_	0.11	*0.58*	−0.26	−0.29	−0.17	0.09	0.14	−*0.40*	*0.91*	
AOX	*0.73*	0.07	−0.29	−0.12	−0.27	*0.68*	0.12	−0.36	−0.19	
GA	−*0.45*	−0.12	0.21	−0.34	0.27	−*0.40*	−0.26	*0.42*	−0.17	
CGA	0.05	0.05	−0.11	−0.01	−0.01	−0.11	0.01	−0.11	0.27	
CA	−0.16	0.11	0.04	*0.98*	0.24	−0.12	−0.02	−0.01	0.23	1
*p*CA	−0.22	−0.07	0.09	−0.38	0.19	−*0.57*	−*0.38*	*0.64*	−0.05	−0.20
FA	−0.34	*0.50*	−0.05	*0.81*	0.18	−0.22	−0.12	−0.10	*0.54*	*0.66*

The statistically significant (*α* < 0.05) correlation coefficients are printed in italic

*∑ Fl* total contents of flavonoids, *AOX* antioxidant activity, *P*–*PO*_*4*_ phosphate phosphorus, *GA* gallic acid, *CGA* chlorogenic acid, *CA* caffeic acid, *pCA p*-coumaric acid, *FA* ferulic acid

In the case of essential elements, their interrelations are affected by participation in biochemical metabolic pathways of medicinal plants. Also, positive relation between total flavonoids and antioxidant activity of the infusions of medicinal plants can be explained by chemical properties of these compounds. A characteristic finding is the high correlation between the total P and its water-extractable inorganic phosphate P form, which confirms our earlier results [13, 14].

### The Essential Elements and the Human Health

Based on the values of the Dietary Reference Intakes (DRIs), it is possible to calculate the contributions of P, Fe, Zn, Mn, and Cu water-extractable forms to the human diet [24]. Assuming that the consumers at the age interval of 51–70 years drink daily two cups of herbal tea (infusion), the amounts of the studied elements were related to the DRIs, as shown in Table 5.

**Table 5:** Contributions of essential elements in herbal infusions to the Dietary Reference Intakes (DRIs) values

Infusion of herbal remedy	Contribution of essential element to DRI (%)						
	P	Fe	Zn		Mn		Cu
	M = W	M = W	M	W	M	W	M = W
*Equiseti herba*	1.3	9.0	1.2	1.7	5.9	7.5	4.5
*Polygoni herba*	1.8	24.1	1.5	2.0	14.9	19.1	0.7
*Hyperici herba*	2.1	5.2	1.7	2.3	26.3	33.7	0.8
*Crataegi folium et flos*	2.1	10.9	1.6	2.2	16.1	20.6	1.1
*Sambuci flos*	4.2	11.9	1.5	2.1	13.2	16.9	1.2
*Chamomillae flos*	3.9	12.1	1.5	2.0	11.9	15.2	1.5
*Helichrysi inflorescentia*	2.0	6.9	2.8	3.8	27.3	34.8	1.8
Mean value	2.5	11.4	1.7	2.3	16.5	21.1	1.7

DRIs for *P* = 700 mg/day (men and women); Fe = 8 mg/day (men and women); Zn = 11 mg/day, men = 8 mg/day (women); Mn = 2.3 mg/day (men), 1.8 mg/day (women); Cu = 0.9 mg/day (men and women). Values for both sexes at age interval 51–70 years

*M* men, *W* women

Inspection of these data has shown that the highest contribution of all investigated essential elements was that of Mn, especially for the infusions obtained from *H. herba* and *H. inforescentia*. Also, the infusions prepared from *P. herba* deliver high, as compared with the remaining herbal infusions, quantities of Fe. These data are in general agreement with the results of previous studies on contributions of Fe, Mn, Cu, and Zn in an infusion obtained from one of the Amazonian plants used in medicine, especially in the case of water-extractable forms of Mn and Zn [25].

### Cluster and Principal Component Analyses

**Fig. 2:**
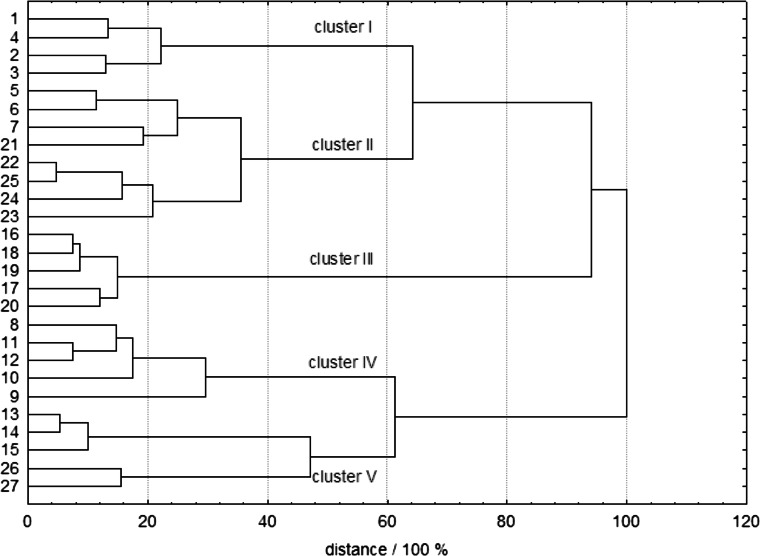
Results of cluster analysis for the medicinal herb samples

The results of CA shown in Fig. 2 allow to distinguish five well-separated clusters grouping the samples with similar properties. Cluster I, which is well separated from the other three clusters, includes almost all samples of *E. herba*, whereas cluster II comprises samples of *P. herba* and *C. flos*. Next, cluster III can be identified as the one with the samples of *S. flos*. The cluster IV contains all samples of *H. herba*, and cluster V comprises the samples from *C. folium et flos* and *H. inflorescentia*.

Preliminary calculations of PCA has shown that when the experimental database comprise all studied parameters (5 phenolic acids, 4 flavonoids, total flavonoids, antioxidant activity, contents of essential elements), the results are not satisfactory because the two first principal components (PCs) explain together less than 50 % of variability among the samples. However, when the starting database contains only 12 parameters, the two first PCs explain together almost 56 % of variability.

**Fig. 3:**
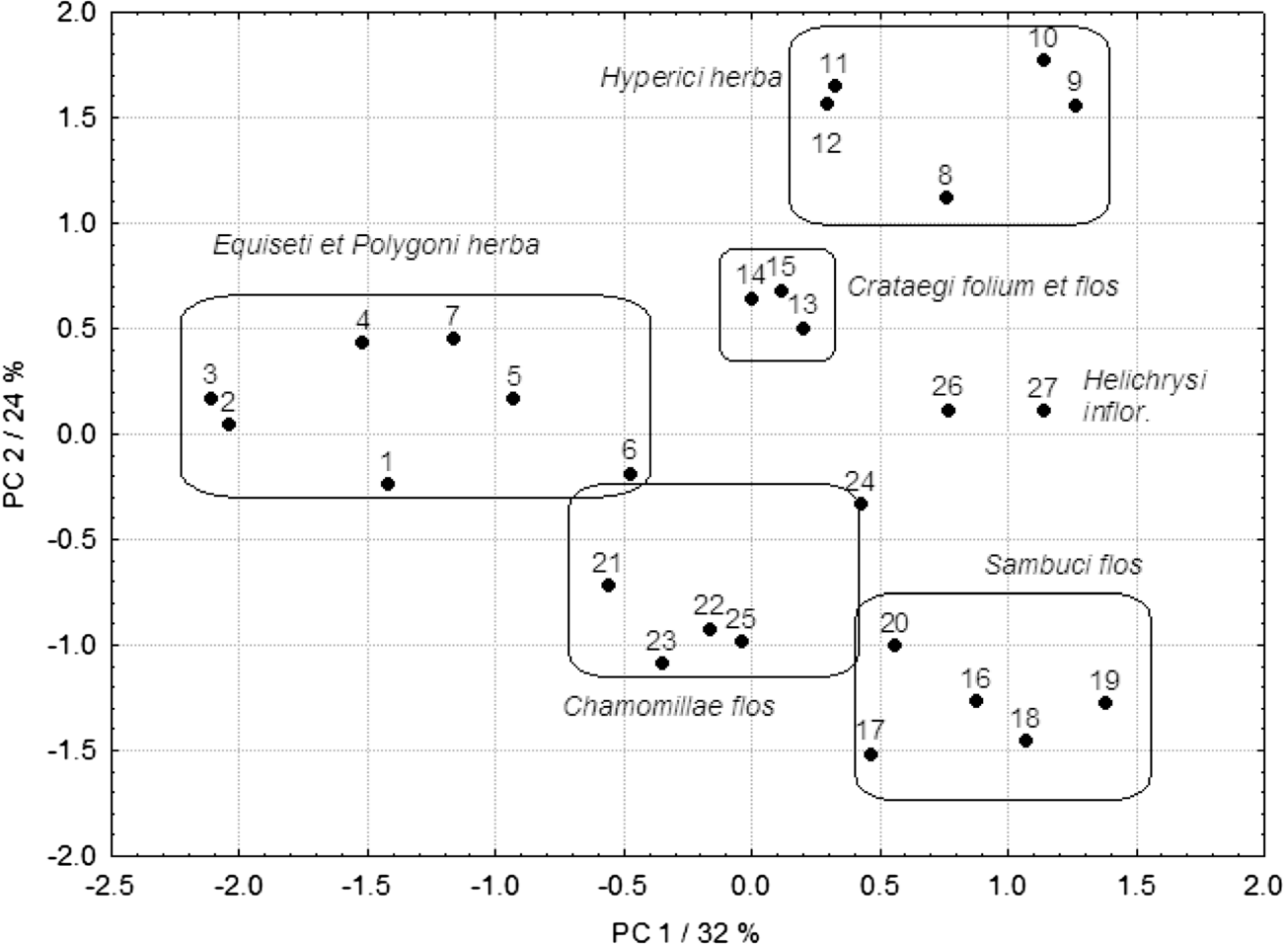
PCA scatterplot of first two principal components obtained for the European medicinal herbs

Therefore, after calculation of PCA, the two-dimensional PC1 vs PC2 scatterplot of the studied samples of medicinal plant infusions (Fig. 3) reveals their characteristic distribution. As it can be noticed, there are five clearly distinguished groups of plant materials. In the left hand part of the plot, there is a group of samples belonging to *E. herba* and *P. herba* and in the right hand upper corner, one can notice a group including *H. herba*. In the central area, there are samples of *C. folium et flos*. Two samples of *H. inflorescentia* are located in the right hand part of the plot.

**Fig. 4:**
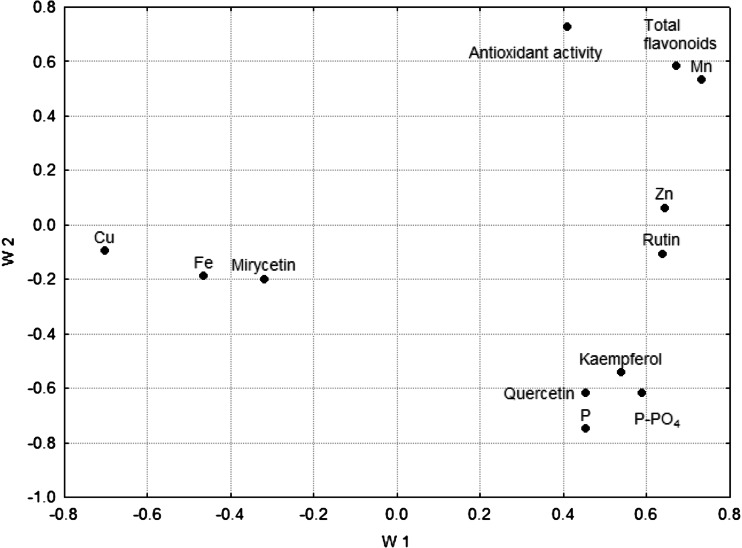
Loading plot for PCA results obtained for the medicinal herbs from different European regions

In the lower part of the plot there are two groups of samples. The first one includes *C. flos*, and in the right hand down corner, there is a second group with the samples of *S. flos*. Thus, it can be stated that the results of PCA are compatible with those obtained by CA. Owing to application of CA and PCA, a massive impact of botanical species of a medicinal plant (genetic factor) could be revealed on classification of the samples. The fact that the studied plants originated from distant geographical areas of Europe was not statistically significant. Moreover, PCA enabled to select the concentrations of essential elements, such as Mn and Cu, also the total P and water-extractable phosphate P, as the factors which strongly influence the differentiation of the studied medicinal plant materials (Fig. 4).

## Conclusions

Based on the study of medicinal plant infusions, the studied plant samples can be characterized by specific phenolic compounds and antioxidant activity along with the contents of selected essential elements. The main conclusion is that plants belonging to different botanical species differ significantly in their chemical composition, as it could be demonstrated by the ANOVA. In general, with the exception of a significant difference in Fe and Zn levels between the samples from Portugal and Serbia, the plant materials do not exhibit statistically significant differences associated with the origin from distant areas of Europe. Moreover, the use of CA and PCA confirmed the massive impact of botanical species of a medicinal plant (genetic factor) on classification of the samples. The analysis of contributions of essential elements to the DRIs indicates that high concentrations of water-extractable forms of Mn and Fe are found in infusions obtained from *H. herba* and *H. inforescentia*.
